# A Computer Controlled Data Acquisition System for Combustion Calorimetric Experiments

**DOI:** 10.6028/jres.093.011

**Published:** 1988-04-01

**Authors:** T. J. Buckley, J. M. Rukkers

**Affiliations:** National Bureau of Standards, Gaithersburg, MD 20899; Department of Informatica, Twente University of Technology, Enschede, The Netherlands

**Keywords:** combustion, computer application, data acquisition, flow calorimetry, modular programming, multiprogramming

## Abstract

At NBS a data acquisition system for a flow calorimeter which accommodates large samples has been developed. The system is based on an instrument controller, scanners, and voltmeters, all available commercially. Detectors for temperature, gas flow rate, pressure, and gas chemical composition provide data on key operating parameters of the calorimeter. A real-time, multi-tasking, general-purpose, data acquisition program is described. Computer science concepts important to the design of the program are explained. The software is driven by tables of data loaded at the start of the experiment. Thus, program execution is changed by providing different tables of information for the data channels desired, data display, and data storage. Tasks for data acquisition, instrument control, data storage, calculations, data display, and run-time-parameter entry are activated or deactivated during the experiment by the operator. Sample results are presented to illustrate the use of the data acquisition system. The software developed for this system is well suited for the changing experiments commonly encountered in the research laboratory.

## Introduction

As experiments become more sophisticated, the demand on data recording increases. This problem is compounded in the research laboratory where instrument development is occurring. As the instrument evolves, the data recording needs change, which often requires hardware and software changes in a computerized system. The following text describes our approach to minimizing the changes needed to cope with instrument modifications.

The National Bureau of Standards has developed a large sample flow calorimeter for use in studies on municipal solid waste [1,2]. This instrument has been used in heats of combustion studies [3] and in investigations on the fate of chlorine during combustion [4,5]. Details of the design and operation of the multi-kilogram capacity flow calorimeter used in these studies can be found in the publication by Churney and coworkers [6].

Calorimetric experiments have long been recognized as being tedious, time consuming, and suited for automation. Westrum [7] has discussed the benefits of automating calorimeters, described the data acquisition system for his heat capacity calorimeter, and listed other laboratories which have automated their experiments. In certain respects our calorimeter is even more demanding on the need for and requirements of data acquisition. As a result, our flow calorimeter has been automated to assist in monitoring its operation and help improve the integrity of the data.

As in any computer controlled experiment, it is both the hardware and software which make the system work. We have assembled a number of hardware components from commercial vendors resulting in a data acquisition system which has proven to be powerful and adaptable. Since the software is what really gives a computer system its operating character, we have taken an interdisciplinary approach, one joining computer science and physical chemistry, to produce a data acquisition system which is suited to our laboratory needs. Our instrument is a research calorimeter and combustor in which the experiments change regularly as we acquire new detection instruments and new insights into the problems being studied. If we are to progress with the changing detector configurations, a software design is needed which is well structured and easy to modify.

In developing the software we resisted the temptation to design a system which was unique to our calorimeter. Rather, we strived to design a software package which was based on the “generic” character common to experiments in general. Only the unique aspects of our calorimeter were treated in a manner which deviated from the “generic” software philosophy.

One of the authors is a computer scientist while the other is a physical chemist. Since this paper is most likely to be read by physical scientists with limited backgrounds in computer science theory, a portion of the following is a primer on concepts important to structured and concurrent programming related to our program. Other sections discuss calorimetry and combustion concerns, the data acquisition hardware and software, and key features of our data acquisition program.

### Flow Calorimetry

The large sample flow calorimeter is unique and has certain data requirements not found in other calorimeters. One feature which is unusual is the sample size which it accommodates. Unlike conventional bomb calorimeters which use sample sizes of one or two grams, our instrument was designed for 2.5 kg samples and has been used with samples weighing up to 5.0 kg. When burned, these samples release on the order of 17 MJ/kg of energy at an average power of up to 35 kW. If the combustion proceeds too quickly, there is not enough heat capacity in the combustor to prevent its temperature from rising to the melting point of stainless steel. As one might guess, this is an undesirable situation. To prevent the possibility of the experiment getting out of control, the combustor is instrumented with an array of thermocouples used to monitor its temperature profile.

The size of the sample is the primary reason for using the flow calorimeter design instead of the conventional bomb design, because of the safety problems associated with a large constant volume system. Unlike the bomb calorimeter, where there is no mass flow between the combustion zone and the surrounding environment, the flow calorimeter requires a substantial volume of gases to enter and leave the combustor. The use of the flow combustion technique for burning the sample necessitated the use of certain detectors (to measure gas flow rate, pressure, and composition) not found on a bomb calorimeter.

Corrections to the observed temperature rise in a bomb calorimeter are about 2% for heat exchange with the surroundings and a total of 0.1% for the difference between experimental and standard states, ignition, and acid formation. The combined corrections amount to about a 2.1% change in the enthalpy of combustion computed from the observed temperature rise alone.

In flow calorimetry, oxygen or air enriched with oxygen enters the combustor and the product gas leaves with varying amounts of oxygen, carbon dioxide, water, and a small amount of carbon monoxide. Thus, the composition and flow rate of the gas must be monitored in real-time during the burn. Corrections to the observed temperature rise are about 4% for heat exchange with the surroundings. Tens of thousands of liters of gas flow through the calorimeter. As a result, corrections due to carbon monoxide formation, water evaporation, and gas heating can be as high as 1.5%. The combined corrections amount to about a 5.5% change in the enthalpy of combustion obtained from the temperature rise of the calorimeter fluid [6].

#### Combustion

Combustion experiments, as distinct from flow calorimetric experiments, place a further burden on the data acquisition system. In combustion experiments, the important questions involve quantitative accounting for the major components and the production of trace amounts of unburned hydrocarbons, chlorinated organic compounds, carbon monoxide, and other species. Some of the species, such as chlorinated organic compounds, are in such small amounts that they must be trapped so a detectable amount accumulates. Other species, such as sulfur dioxide, are produced in large enough quantities allowing them to be observed using real-time standard spectroscopic techniques. The nature of the experiments and the calorimeter/combustor requires many channels of data to be recorded for extended periods of time.

### Experiment Sequence

The calorimeter/combustor can be operated in two different modes: either as a combustion calorimeter or just as a combustor. When operated as a calorimeter, the temperature of the calorimeter water is most important. The compositions, temperatures, and flow rates of the inlet and outlet gases are of secondary importance. When used as a combustor, the primary parameters are the secondary ones for the calorimeter plus the combustor temperatures.

Table 1 lists the sequence of events during the two modes of operation. After assembly, the calorimeter is allowed to attain equilibrium heat exchange with its surroundings during the initial temperature drift period. The combustion zone is then purged of air with the oxidizing gas. The sample is ignited, followed by the burn, then the combustion zone is purged of product gases. The final drift period, instrument calibration, and disassembly complete the experiment. The sequence of events are similar for combustion experiments differing by the absence of drift periods before and after the burn and the final purge. Due to the size of the calorimeter, the time needed to reach a final steady state heat exchange with the surroundings is on the order of 2 hours. As a result, a combustion experiment requires less than half the time as a calorimetric experiment. The similarities between the two modes of operation compelled to us to develop generic software.

## Experimental Apparatus

### Calorimeter Configuration

**Figure 1:**
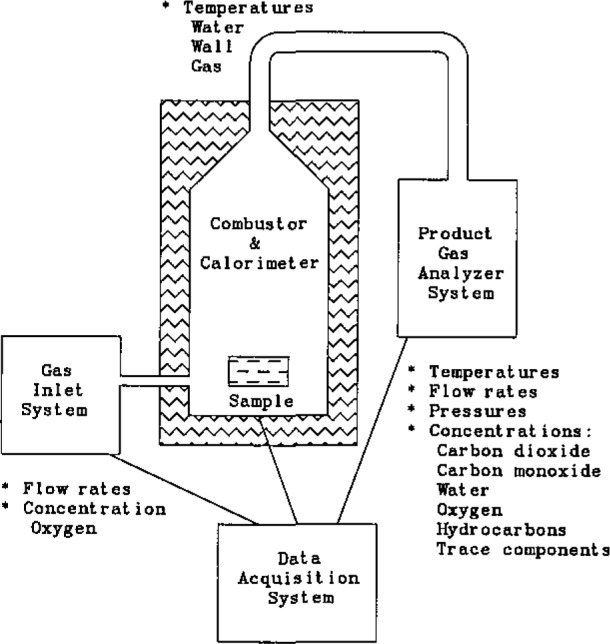
Block diagram of the large sample flow calorimeter and its data system. The parameters recorded from each section are indicated by a star (*). Unstarred items are specific data taken under the parameter listed above them.

The large sample flow calorimeter is diagrammed in figure 1. There are three main parts to the calorimeter: the gas inlet system, the combustor and calorimeter, and the product gas analyzer system. These sections are interfaced to a data acquisition system which records and displays the incoming data in real-time.

The volume and composition of the gas entering the combustor is controlled by the gas inlet system. The valving in the manifold allows one to control the oxygen content of the oxidizing gas, monitor its flow rate, and intercompare the flow meters.

The calorimeter and combustor section is made up of a stainless steel combustion chamber with gas preheaters and six tiers of inlet nozzles that direct the oxidant gas at and around the sample. The surrounding calorimeter contains a metric ton of water which absorbs most of the heat of combustion. This section of the instrument is equipped only with temperature sensors.

The product gas analyzer has two sections. One is a gas trap for collecting chlorinated organics used during the fate of chlorine experiments. The other, used in every experiment, is composed of a group of instruments which measure the exhaust gas characteristics. They consist of infrared detectors, pressure meters, temperature transducers, and a dew point meter to mention a few of the instruments involved.

### Detectors and Sensors

Table 2 lists the various sensor types used to monitor the operation of the calorimeter. There are four classes of detectors and sensors used in our experiments: temperature, gas flow rate, pressure, and gas composition. The gas inlet system uses differential mass flowmeters [8] and a rotameter [9] to monitor the air and oxygen flow rates into the combustor^1^. These meters generate analog voltages with full scale readings corresponding to flow rates of 15 to 500 liters per minute depending on the meter. A ZrO_2_ EMF cell [10] monitors the inlet oxygen content.

The calorimeter/combustor assembly is equipped with temperature sensors of Type T and Type K thermocouples. The Type T couples are used to monitor temperatures near room temperature such as the gases entering and leaving the calorimeter while the Type K couples are used to measure high temperatures such as those of the combustor walls. Two quartz crystal thermometers [11] are used to measure the temperature of the calorimeter and surrounding jacket water.

The product gas analyzer system involves several detector types. The temperature of the gas at various points is measured by Type T thermocouples. The concentration of hydrocarbons on the ppm level is monitored by a hydrocarbon analyzer [12] whose flow rate is measured by a rotameter [13]. Water vapor is measured by a cooled-mirror humidity analyzer [14] while the CO_2_ and CO concentrations are determined by dedicated infrared detectors [15]. Minor constituents, such as HCl and SO_2_, are monitored by a scanning infrared detector [16]. The gas pressure in the analyzer system is measured at three points by using an absolute manometer [17] and two differential manometers [18]. A ZrO_2_ EMF cell [19] measures the product gas oxygen content. The chlorinated organic trap is instrumented with Type T thermocouples for temperature monitoring.

These detectors have a variety of different outputs from microvolt level signal from the thermocouples, to five volt signals from the flowmeters, to frequencies from the quartz crystal thermometers, to serial data (ASCII characters) from the scanning infrared spectrometer. In order to accommodate this wide range of voltages and data types, a versatile data acquisition system is needed.

### Data Acquisition System

Until the purchase of our current instrument control system, we used a microcomputer and a minicomputer to record data from our experiments. The limited memory sizes (64KB, kilobytes, of RAM) and processing capabilities of these machines quickly became obstacles in our effort to expand the productivity of the data acquisition system. The problems were further complicated by the great differences between the operating systems, programming languages, and utilities of the two computers. These limitations have been remedied by our current system which replaces the two computers previously used.

**Figure 2:**
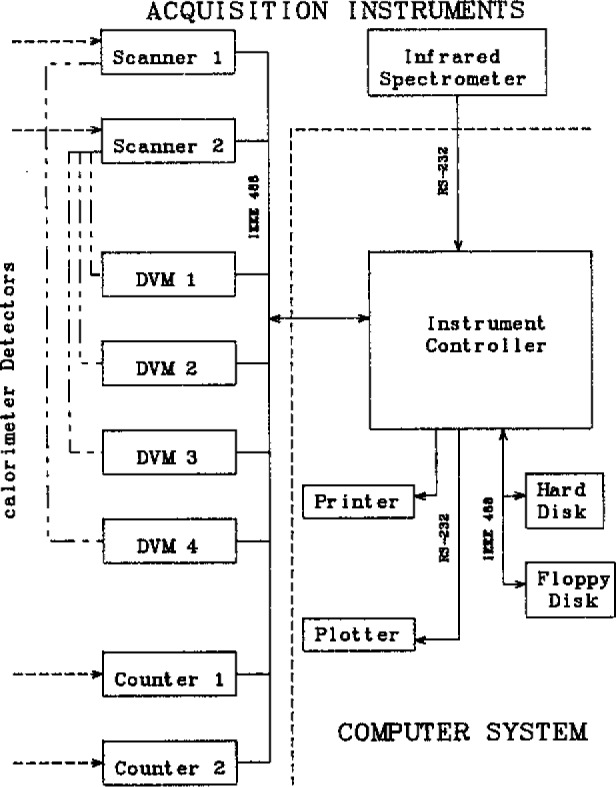
Diagram of the calorimeter/combustor data acquisition instruments and instrument control system.

A block diagram of the data acquisition system is shown in figure 2. The system can be divided into two parts: the acquisition instruments (top and left) and the computer system (lower right corner). The choice of the acquisition instruments allows for flexibility in the handling of the various detectors and sensors. Signals from most detectors arrive as analog voltages at two scanners [20] which provide, at present, a total of 80 channels of input. Of the 80 pairs of switches making up the two scanners, 20 have less than one microvolt thermal offset while 50 are rated at less than two microvolts. These switches are used for measuring single thermocouple voltages to obtain a temperature resolution of a tenth of a degree. The remaining 10 pairs of switches are rated at 30 microvolts and are used only for high level signals. These switches allow us to handle a wide dynamic range in analog signal input.

The signals from the scanners are sent to one of four high precision digital voltmeters (DVMs) [21] to be digitized. The auto ranging feature of the DVMs allow the recording of signals ranging from a microvolt to a thousand volts. Two counters [22] are used to measure the frequency of the quartz crystal thermometers. The voltmeters, counters, and scanners used are well suited for the research laboratory by allowing one to measure ac/dc volts, resistance, or frequency with wide dynamic range and high precision.

The scanners and voltmeters are interfaced to the computer system through an IEEE-488 communications bus. The scanning infrared spectrometer sends data to the computer system through an RS-232 serial communications port.

The data acquisition system is controlled by an instrument controller [23], a unit which has also been used by Gallagher [24] in a system to acquire data from calorimeters. This computer is designed to service communication buses and ports in applications where the data acquisition instruments generate interrupts. The computer has 136KB of RAM of which 64KB is available for the operating system and user software. A high resolution monochrome graphic display with a touch sensitive overlay provides a convenient user interface. Mass storage is provided by two 400KB floppy disk drives, a 10MB (megabytes) hard disk drive, and 512KB of RAM used as a disk. Hard copy of text and graphics is provided by a dot-matrix printer and a multi-pen color plotter. An IEEE-488 and four serial communications ports allow the computer to access the instruments.

### Software Tools

The Fluke instrument control system is furnished with several software tools and language features. Fluke’s own disk operating system (FDOS) provides basic control of the hardware. A self test is performed upon power up while a diagnostics package aids in hardware trouble shooting. An editor, file manager, and other utility programs are provided for system maintenance.

The computer can be programmed in any of four languages: FORTRAN, Interpretive BASIC, Compiled BASIC, and assembly language. A linker allows limited combinations of the four languages, such as subroutines written in FORTRAN can be executed by a program written in Compiled BASIC.

While Interpretive BASIC is useful for testing new hardware devices and program segments, Compiled BASIC is used for execution of our calorimeter program due to its faster execution speed. Other features, such as the use of true subroutines and flow control statements, help in software development. Fluke’s Compiled BASIC has program flow control statements (EXTENDED IF, WHILE, REPEAT, LOOP, LEAVE, and SELECT-CASE) found in Pascal, a language designed to teach programming as a systematic discipline. Virtual arrays, arrays used as if in main memory but reside on disk, greatly expand Fluke BASIC’s ability to handle large data sets. The program also can be made much larger by making sections of the code into overlays which are stored on disk and only loaded when needed. The instrument control character of the computer is evident in the high level language structures provided for servicing interrupts. Interrupts can be called by a serial port, an IEEE-488 port, time of day and interval clocks, an error, or a touch sensitive screen. These and other features make the system very useful in a real-time interrupt-driven environment.

## Data Acquisition Software

### Structured Programming

The demands of large sample flow calorimetry, the idiosyncrasies of the hardware used, and the flexibility needed in a research laboratory environment dictate a software package which is not trivial. When developing complex software it is very important to use a structured approach to programming. This is especially true when working with languages such as BASIC which have arbitrary transfer of control statements (i.e., the GOTO statement).

An unstructured approach may seem expedient, since there will finally be a program that “gets the job done,” but in the long run this approach is uneconomical due to the time spent in later modifications of the software. Unstructured programs very often turn out to be “logical spaghetti” which are not easy to modify or debug. Computer scientists such as Dijkstra [25] have discussed in detail structured programming and its relationship to effective and reliable programs. Based on the advice of the experts, we proceed with the structured approach to programming.

### Computer Science Concepts and Definitions

When developing a program, a design methodology is needed. A very good and widely understood method is the top-down approach. This means that one first comes up with a general description of the overall program structure, and then starts refining the various parts until the actual program is generated. Starting with a mixture of natural language type descriptors and formal control functions (WHILE, REPEAT, etc.) refinements are made until the detailed program statements are produced.

For the interested reader, more details concerning these topics can be found elsewhere. The following lists some excellent papers dealing with topics of interest: structured programming and correctness, hierarchical program structures, structuring of data (which has a significant impact on the program) [26], top-down programming [27], managing of program development [28], and concurrent programming [29].

The introduction of the concept of modular program code is very important when structuring a program. Program modules introduce a separation of concerns: different processes reside in different modules. This concept can be applied at many levels of abstraction [26,28]. Modular programming is a great thinking and development aid and promotes the design of a well structured system.

### Processes

A sequential program specifies sequential execution of a list of statements; its execution is called a process. A concurrent program specifies two of more sequential programs that may be executed concurrently as virtual parallel processes.

A concurrent program can be executed by allowing processes to alternately share one processor. This approach is referred to as multiprogramming and is supported by an environment that multiplexes the processes on the processor [30]. The program operates as if each process is executed on its own virtual variable-speed processor.

In order for processes to cooperate, concurrently executing processes must communicate and synchronize with each other. Communication allows execution of one process to influence the execution of another. Interprocess communication is based on the use of shared variables (data that can be referenced by more than one process).

Synchronization is often necessary. Since processes are executed with unpredictable speeds, one process may have to wait for certain actions to be performed by another process before continuing its execution. For example, imagine a process that is acquiring data from an external source. Another process, that performs an operation such as a calculation using the data from the former process, will have to wait for this process to make the data available before it can do its computation.

Processes can be divided into two categories: foreground and background. Background processes are either active or passive (dormant). When a process is activated, it is inserted into the background and executes until it becomes passivated. More than one background process can be competing for the computer’s resources at a given time. Foreground processes are essential interrupt-driven operations. They have priority over the background processes, whose execution is preempted to make processor time available for the foreground process. Foreground processes cannot preempt one another.

In order to multiplex the various processes on one processor, the following scheduling mechanisms are provided. The foreground solution is straight forward; the occurrence of an interrupt is the scheduling entity and since only one interrupt can be active at a given time, no other mechanism has to be provided.

The background processes are scheduled by using the following continuous loop solution.

REPEAT

 IF active(p1) THEN p1 (interface list)

 IF active(p2) THEN p2(interface list)

 .

 .

 IF active(pn) THEN pn(interface list)

UNTIL False

The boolean variables used in the loop, active(pn), are guards signalling the state of a process, pn, being either active or passive. When a process is activated, the subroutine for the process, pn(interface list), is called. Thus, the processor time is divided over the active processes. If there are no processes active, then the computer waits in the loop until an interrupt occurs at which time one of the processes may be activated.

A scheduling mechanism should be fair to be adequate, e.g., no process should be delayed forever. The continuous loop described is bounded fair. That is, a process waiting to get access to the processor is delayed for at most the execution times of the other active processes. Of course, the continuous loop scheduling mechanism is fair only when none of the other processes is executing indefinitely. To insure this is so, it is desirable that the execution time of a process at each call is “short.” The continuous loop scheduling mechanism will then establish a good sharing of the processor time. For a more complete discussion of fairness see the article by Lehman [31].

When dealing with concurrent processes, it is necessary to insure that no unwanted influence will take place between two processes. There will be some critical sections of code that have to be protected. This is partly provided by the BASIC language used since it executes statements on one line in an atomic matter; interrupts can only be serviced before or after the statement is executed and never during. The language also provides ENABLE and DISABLE interrupt statements that can be used to protect critical program segments from unintentional changes. These entities give sufficient possibilities for solving the unwanted influence problems we faced.

The possibility of introducing a deadlock situation has to be considered. Deadlock is a state of affairs in which two or more processes are waiting for events which will never occur. More explicitly, a processes will continue testing a certain variable for change but will not see it (this is sometimes called “spinning”), because the process that is supposed to set this variable is blocked on a variable that has to be set by the first process. This situation has to be prevented from occurring by carefully using the continuous loop scheduling mechanism.

The continuous loop scheduling mechanism prohibits very long iterations or recursions in a process using the WHILE or REPEAT Compiled BASIC language commands. In order to iterate, the process must save its variables upon completion of any iteration step and continue on the next opportunity through the scheduling loop. The mechanism used for saving the state conditions and for holding the shared variables used for synchronization is called the interface list. It consists of variables that are exchanged between the process and the main program and used for the previously mentioned purposes. The interface list is explored in more depth in the following section.

#### Program Environment

A process needs an environment in which it can be executed. This environment consists of two parts: 1) a dynamic environment formed by the behavior of the active processes, 2) a static environment consisting of the data structures that are used to enable the process to be aware of the dynamic or changing environment.

The static environment can be viewed as a window to the state of the system at a particular moment. This window is provided by the parameter lists of the processes. By examining the variables of the parameter lists, the program determines what the appropriate actions are under the given conditions. Thus, this list is essentially an interface list that couples the necessary global data to the processes.

Using the static environment, it is possible to synchronize the various processes by letting the interface lists overlap, thus providing variables common to the interrelated processes. It should be stated that, while this solution to the synchronization problem is sufficient, it is not the best way to solve the problem. It would be convenient to use some more sophisticated primitives for scheduling and synchronizing to give one more flexibility and a better program structure. The P&V concept, the cobegin [30], and the fork and join [32] are worth mentioning in this context. The overlapping interface list solution is considered the best possible, given the limitations of Compiled BASIC, and provides a good and conceptually clean program structure.

#### Modules

**Figure 3:**
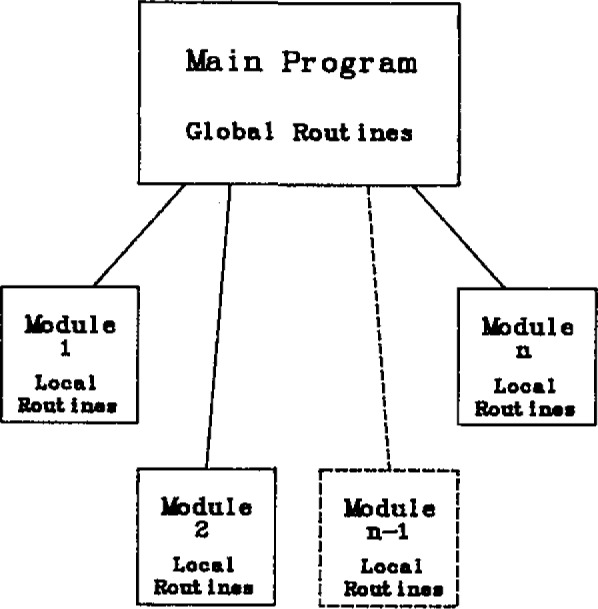
Diagram of the calorimeter program architecture showing the creation of modules as subsections of the main program.

As previously stated, a modular approach is desired to control the complexity of the system. It also provides a separation of concerns by localizing entities to the processes where they are used. Essentially, a module consists of what we call a process and the local routines that support the execution of that process. Since there are also the global routines that make up the environment of a process, the overall architecture can be pictured as in figure 3. The main program, which manages the scheduling of the processes, is coupled through interface lists to the modules, which contain the computer commands for the tasks discussed next.

#### Experiment Tasks

The structure of our data acquisition software is developed from the combustion and calorimetric experiment task list. The tasks identified as characteristic of a general purpose combustion experiment are given in table 3. In order to implement the program design, the interrupt-driven tasks are identified as the foreground processes. They involve the data collection, the data channel selection, and error handling. The rest of the tasks are implemented in the background processes using the continuous loop scheduling mechanism previously described. The processes to be performed can easily be written in the form of modules to complete the program structure.

### Program Configuration

An important feature to support the flexibility of our program is the program database. Since the data acquisition sequence may be changed very often, the program has the opportunity to be tailored to the required data channel selection scheme. The data acquisition sequence is handled by a timetable database which can be easily changed or generated. Therefore, a change in the acquisition sequence does not involve recoding but uses the features of a database generator to create the desired data structure. Also, the displays are of a general character, driven by a display database structure that also can be easily changed. Our program for data acquisition is divided into three parts: the program statements, database, and database manipulator.

**Figure 4:**
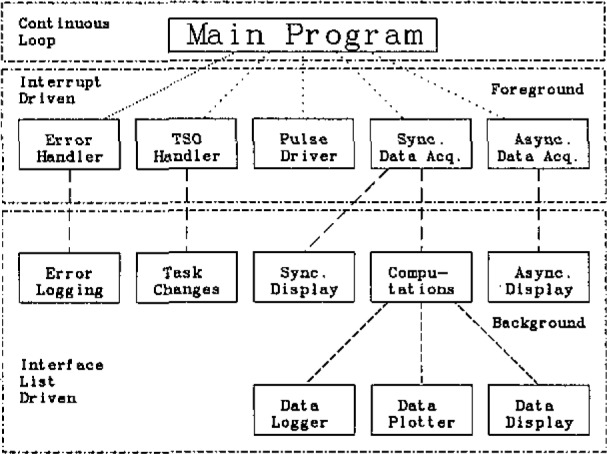
Total architecture of the calorimeter program. The main program operates in a continuous loop. Interrupts force various processes to be performed, then pass on parameters in an interface list to enable execution of other processes.

Figure 4 shows the overall architecture of the cooperating processes which make up the data acquisition program. Shown are not only the real processes as they were derived from the initial task recognition and analysis, but also how processes are synchronized (indicated by the dashed lines) relative to one another. As an example, the process displaying the results computed from the synchronous data has to first wait for the data to be acquired and computations completed. The interface list driven processes are called from the continuous loop of the main program while the interrupt driven processes are executed when an interrupt occurs.

### Data Acquisition

The data acquired can be separated into two different types: synchronous and asynchronous data. The acquisition of synchronous data is controlled by the clock and composes the majority of the data recorded during our experiments. The asynchronous data arrives at the computer at a pace which is not dependent on operation of our data acquisition program but only upon the instrument transmitting the data.

#### Synchronous Data Acquisition

The scheme we use for the acquisition of synchronous data can be viewed as an active foreground process. This process is constantly executing a data logging loop in which channels are selected for digitizing at a specific moment. The timing of the data acquisition scheme is interrupt driven, so that processor time not used when the acquisition process is idle can be used by active processes in the background.

Our data logging loop is established by a one second interrupt interval timer we call the experiment “pulse.” Each second, the timer generates an interrupt which causes program statements in the pulse driver process to be executed. The pulse driver uses the timetable database (described later) to determine which channels should be selected by a scanner during a particular second and triggers the data acquisition devices attached to the channels. When the devices have their data available, they generate a service request flag and the background processes are temporarily stopped to allow the synchronous data acquisition process in the foreground to be executed.

#### Asynchronous Data Acquisition

The acquisition of asynchronous data is a foreground process in which our program does not initiate the data coming in but only records it. This mode of operation is characteristic of data from our scanning infrared detector, which sends data as it becomes available down a serial interface line to the data instrument controller. Datum from the serial port generates an interrupt which is then read and stored on disk by the asynchronous data acquisition process if it is activated, otherwise the data is lost.

#### Timetable Database

The foundation of our data acquisition program is formed by the timetable database. The information stored in this database is used for determining the behavior of the program. The timetable gives the program the channel numbers to be selected at a specific time and the device from which to read the data.

**Figure 5:**
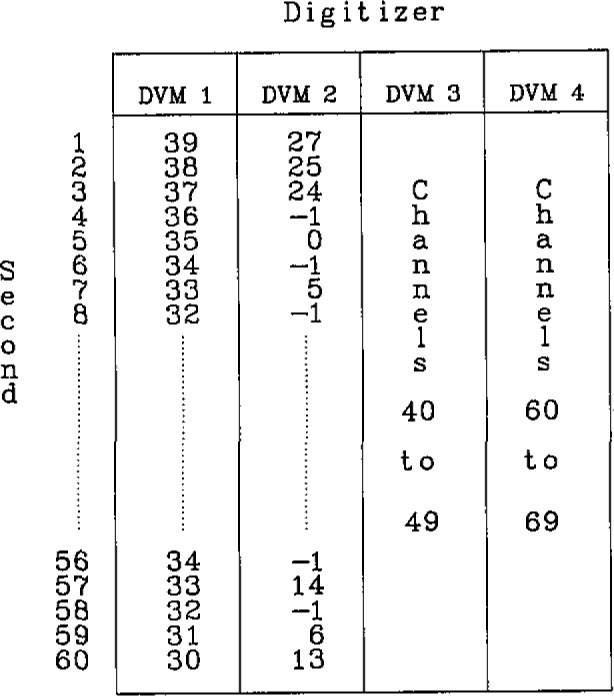
Two-dimensional timetable used to sequence the data acquisition channel selection. Each row corresponds to a second in a minute and each column corresponds to one of four DVMs. The non-negative numeric entries are scanner channels to be digitized while the minus ones are when no data are recorded.

The structure of the timetable, shown in figure 5, is closely related to the experiment. A minute is divided into 60 seconds, each in which data collection can take place. The timetable is a two-dimensional array 60 rows long by four columns wide. Each row corresponds to a second of each minute in which any combination of four DVMs can be given a channel to digitize. When a non-negative entry occurs in the timetable, it indicates that channel is to be selected by the scanner and the appropriate device to be triggered. Eventually, the data acquisition device responds by sending a service request flag to the computer, followed by the execution of the synchronous data acquisition process which reads and stores the datum on disk.

The introduction of this timetable concept provides a powerful and flexible tool for tailoring the program to the needs of a specific experiment, a general need characteristic of a research laboratory. It is not necessary to change the program code in order to change the behavior of the program, the only thing to be done is to generate a timetable which resembles the structure of the experiment to be performed.

#### Data Storage Structures

Storage of the acquired data is provided by the implementation of an abstract data structure, consisting of several virtual arrays linked together and the subroutines GET and STORE we developed. This approach enhances the language features that were available in Compiled BASIC by allowing for a single data storage array that is much larger than is normally permitted. Since the programmer only has to use the STORE and GET subroutines to save and retrieve data from storage, there is no problem of finding the data’s location in the linked virtual arrays.

##### Synchronous Data Storage

The data manipulated by the program is divided into two groups: the raw data coming from the data acquisition instruments and the calculated data derived from the raw data. Raw data are saved on a hard disk, thus providing a non-volatile storage medium. This protects the data already recorded from being lost in case the computer system goes down. The calculated data are stored on an electronic disk to provide fast access to the derived quantities.

**Figure 6:**
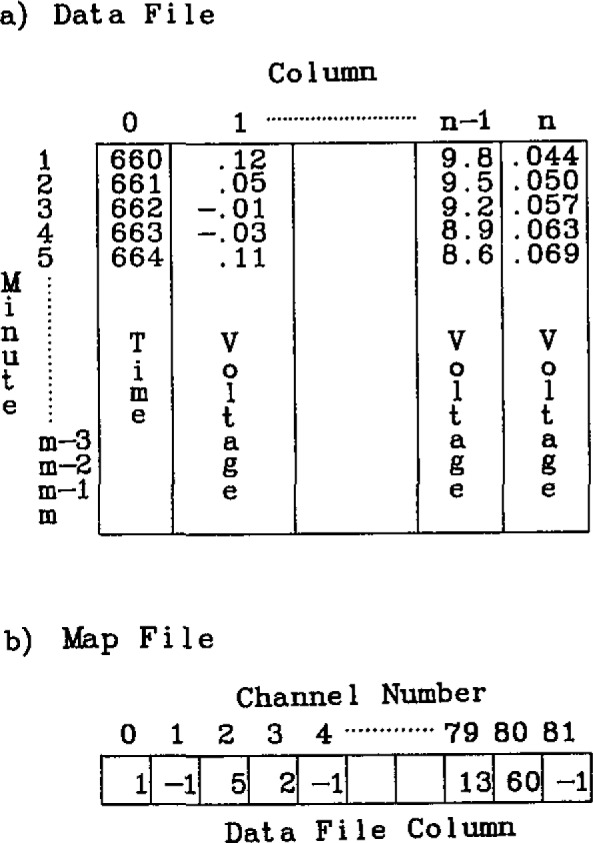
Diagrams of data file (a) and map file (b) used to store synchronous data from the channels selected in the timetable. Each row in the data file corresponds to a minute when data are recorded. Column 0 entries are the minutes in real-time when the data were recorded. The rest of the columns contain voltage measurements corresponding to one of the scanner channels recorded during the experiment. The map file entries are column numbers of the data file used to store each of the scanner channel numbers. A – 1 indicates that the scanner channel was not recorded.

The synchronous data are stored in the equivalent of a two dimensional array of the form shown in figure 6a. This array has the structure of the timetable. Each row of the data array corresponds to a minute during the experiment. Row 1 corresponds to the first minute while the total number of minutes allowed is dictated by the amount of virtual array space allocated and the number of channels recorded. Column 0 of the array contains the real-time in minutes at which the minute started while the remainder of the columns record the voltages.

Due to the data structure, only one value can be stored for a specific channel for a given minute. When multiple readings are taken from a single channel, either the last voltage read or the average of the set of readings is stored.

Usually, only a subset of the available channels are recorded during a specific experiment. In order to optimize storage use, a variable mapping of the channels to the array columns is provided by using a map file illustrated in figure 6b. This one dimension array is 82 (0 to 81) units long. Each entry corresponds to a physical channel number; the first 80 are for the scanner channels and the last two are for the frequency counters. When a channel is to be recorded during an experiment, it is assigned a column number in the map file. If a channel is not to be used, it is assigned a – 1. Note that the channels do not have to be assigned sequentially since the GET and STORE subroutines use the map file for determining data placement.

This technique has a distinct advantage of efficient utilization of storage space. One can trade off the maximum recording time for the number of channels recorded. For every 64KB of storage used (up to 256KB can be assigned to an array) two channels can be recorded for 45 hours while all 80 channels can be saved for 1.7 hours.

##### Asynchronous Data Storage

Storage of the asynchronous data is provided in a fashion similar to the synchronous data. Since there are no channels and minutes, the dimensions of the data storage array is of a fixed length and width. The asynchronous data in our experiment comes from the scanning infrared detector and have a known order. The absorbance of the sample at several wavelengths is monitored, transmitted as serial datum to the computer, and printed in order. Each row of the data storage array is designed to be wide enough to save each set of absorbance readings plus one more column for the minute during which transmission of each data set started. An end of record (the characters carriage return and line feed), which separates the data sets, provides a signal to indicate when the row count of the data storage array should be incremented and the time information recorded.

### Interactive Program Control

Since our program is actually made up of several processes that can be either active or dormant, a tool has to be provided to manipulate the state of these processes. Therefore, we developed operator interface software which exploits the features offered by a video screen with a touch sensitive overlay (TSO). Pushing a touch sensitive pad on the screen initiates the foreground process, TSO handler, which activates or passivates a process. The TSO is also used to update various on-line parameters, such as references temperatures and plotting parameters, entered by the operator.

### Miscellaneous Features

A good deal of the processes are mutually exclusive, that is they do not execute concurrently, so they can be exchanged in and out of memory while occupying the same region of memory. This is possible by using the overlay feature provided by the computer’s operating system. The main program, global routines, and the processes that are frequently executing concurrently always remain in memory. The rest of the processes are mutually exclusive (such as the displays) and reside in overlays and may only be present in memory when the process is executing.

A crucial feature of our program worth mentioning is its ability to recover from errors that may occur. Even when the computer system totally goes down, no data which has already been recorded is lost as long as the computer’s real-time clock is not corrupted. This ability to recover is due to our storing the data on the hard disk immediately after reading them from the data acquisition instruments. When the computer is restarted, our program just continues the data acquisition where it would have been if the data recording had not been terminated. The resulting data set will have a blank spot in it, however, all of the data will be properly timed relative to the first part.

The ability of our program to recover from errors and continue collecting data is made possible by deriving the data acquisition timing from a realtime clock. The instrument controller’s clock is read at the beginning of each system pulse. Subtracting the starting time, which was stored on the hard disk, from the pulse time gives the experiment time. This is converted into minutes and seconds. The minute is used to point to the proper synchronous data storage row, while the second tells which entry in the timetable should be used for channel selection.

## Sample Results

No description of an instrument system would be complete without the displaying of sample results. The initial record of an experiment is the raw data file stored on the hard disk. While the calculated data provide a more meaningful picture of what went on during a burn, they can be regenerated from the raw voltages recorded. Another valuable record is the list of printed data values logged during the burn. This record allows one to readily look up values for various times of the experiment and provides an added level of backup in case the disk files are lost.

**Figure 7:**
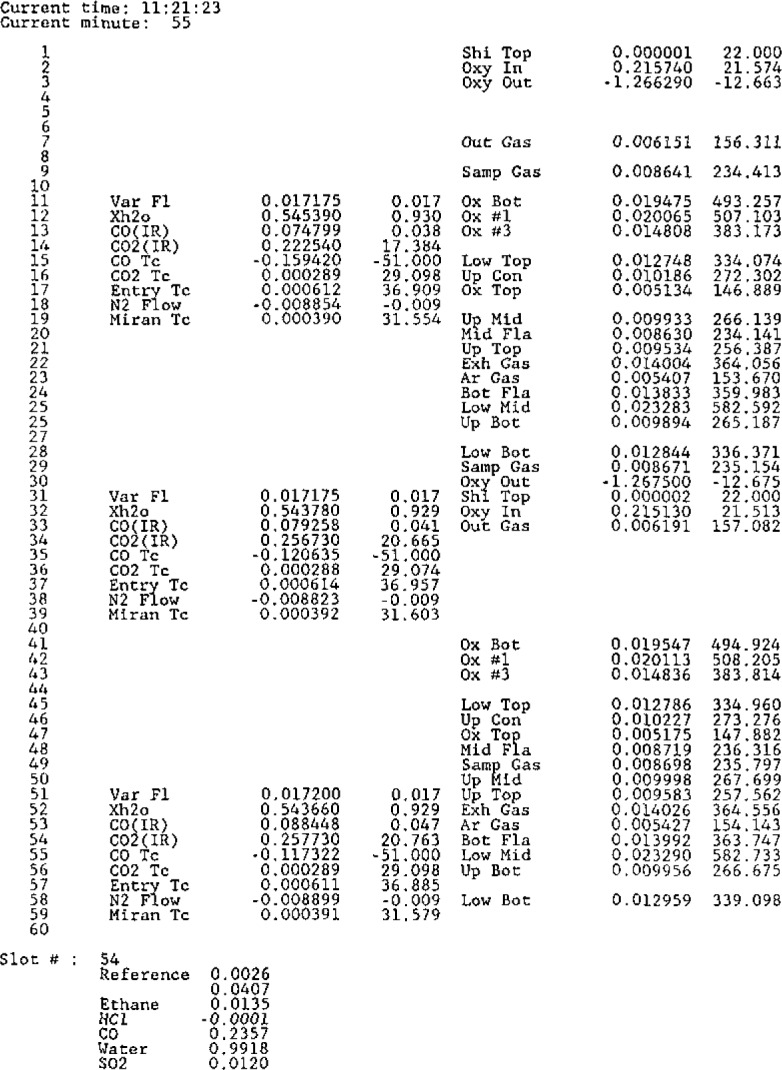
Sample print out from a typical experiment. Data from two DVMs are shown, while the data from the other two have been deleted for simplicity. Each numbered row corresponds to one second during a minute of synchronous data recording. The last eight rows show the asynchronous data storage row (Slot *#*) and the absorbance measurements from our scanning infrared detector.

The left half of one page from an experiment is shown in figure 7. The overall form of the page is similar to the channel selection pattern used. One page is produced for each minute of an experiment. The time of day and the experiment time, in minutes, are printed at the top of each page. The first column contains the second in which the data was recorded, while the next three columns contain a label, raw voltage, and calculated value for data from DVM1. This pattern is repeated for the next three columns for DVM2 and in two more sets of columns for DVM3 and DVM4 (not shown).

The columns for DVM1 show a regular pattern of nine channels digitized three times per minute. The values for temperatures are individual measurements while the flow rates are averaged ones. The columns of data for DVM2 show a repeat of some data but not with the regularity of DVM1. Blank entries in the columns correspond to seconds when data was not recorded.

The bottom of the page has a set of data received from the scanning infrared detector. The data set number (Slot #) is followed by the absorbance readings at seven selected wavelengths. This set of numbers is printed at the next available opportunity after all seven readings are recorded. The timing is usually such that the numbers printed were recorded during the previous minute.

The video display contains the information shown in the printed output but organized in logical groupings of channels. Besides the label for a particular channel, the raw voltage or computed value is displayed along with the change since the last minute. Graphs of a selected channel as a function of time can also be plotted on the screen to see long term trends in the data. The video display also provides menus for the TSO which allow the operator to activate and deactivate various processes.

The use of the data to obtain calorimetric information has been discussed in detail [3] and will not be presented here. However, the use of the data for combustion experiments has not been presented previously and is given in the following discussion.

**Figure 8:**
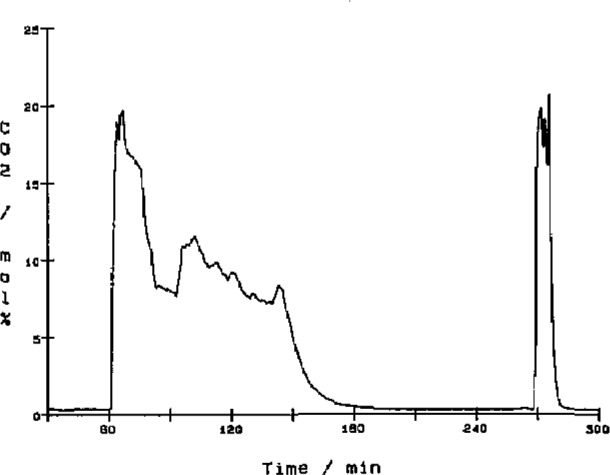
Plot of mole percent CO_2_ data as a function of experiment time. The sample burned during the combustion experiment was municipal solid waste with 5% added lime. The sample was ignited at minute 61 and burned until minute 190. The peak at minute 275 was caused by a 20 mol% carbon dioxide reference gas used to calibrate the detector.

**Figure 9:**
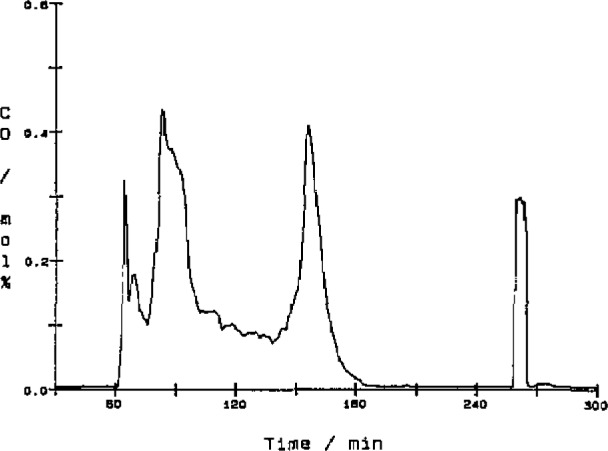
Plot of mole percent CO data as a function of experiment time. The sample burned during the combustion experiment was municipal solid waste with 5% added lime. The peak at minute 260 was caused by a 0.3 mol% carbon monoxide reference gas used to calibrate the detector.

**Figure 10:**
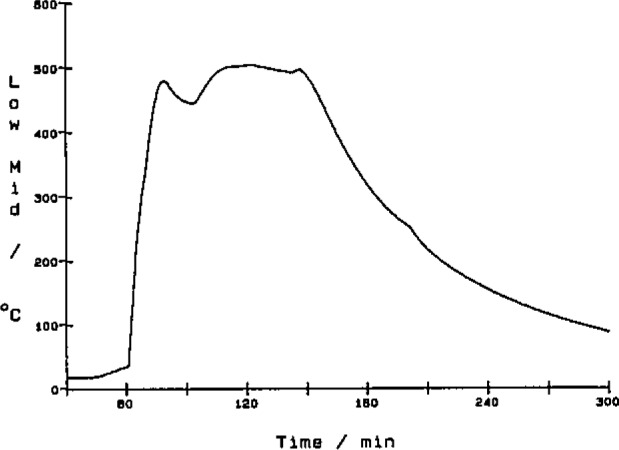
Plot of the combustor wall temperature, approximately 40 cm away from the sample, as a function of experiment time. The sample burned during the combustion experiment was municipal solid waste with 5% added lime.

Figures 8 to 10 show graphs of three channels of data from a typical experiment where a sample of municipal solid waste with 5% added lime was burned. From these data we derive other physical quantities. The CO_2_ (fig. 8) and CO (fig. 9) data are combined with flow rate data to give molar flow rates. The CO_2_ molar flow rate curve is integrated to give the total amount of carbon in the original sample or used as is to indicate the rate of combustion. The CO molar flow rate curve gives a measure of the completeness of the combustion reaction.

The effects of changing parameters such as the gas flow rates or its oxygen content can be observed as changes in the slopes of these curves. The sample was ignited at minute 61 which is marked by a steep rise in the CO_2_ concentration. At minute 71 the oxygen concentration was reduced to slow the burn down, but this was followed by an unacceptably high concentration of CO at minute 82. While watching the computer readouts, the oxygen concentration and gas flow rates were adjusted to lower the CO to an acceptable level of 0.1 mol%. The large CO peak at minute 156 is a characteristic peak that usually occurs when the sample combustion nears completion. The ability to see the data in real time allows us to control the combustor to study the effects of different operating conditions.

Two other peaks can be seen at minutes 262 and 273 minutes into the experiment. These correspond to calibration data for the CO and CO_2_ detectors, respectively.

Figure 10 shows the combustor wall temperature at a point near the burning sample. The wall reached nearly 500 °C for a good part of the burn. This is not quite the 600 °C we desire during a combustion experiment but well below the 900 °C critical temperature where the combustor can be damaged. The break in the curve at 202 minutes is due to the cooling gas circulated around the combustor after the burn. The temperature drops slowly to a level where the combustor can be disassembled.

## Summary and Conclusions

Our data acquisition system has proven itself through real experiments to live up to its design. The hardware provides a versatile foundation for data acquisition. The autoranging capabilities of the DVM’s compensate for differences in signal level while the scanners provide plenty of room for expansion. The modular form of the data acquisition software has enabled us to modify the program with a minimum amount of trouble and debugging. The data tables are easy to modify and let us change the recording and displaying of data to suit the needs of our experiments. The tabular form of the recorded data makes it easy to manipulate and export to other computers for further analysis.

## Figures and Tables

**Table 1:** Comparison of the sequence of events for calorimetry and combustion experiments

	Event	Calorimetry	Combustion
1)	Assemble	X	X
2)	Temperature drift	X	
3)	Purge	X	X
4)	Ignite	X	X
5)	Burn	X	X
6)	Purge	X	
7)	Temperature drift	X	
8)	Calibrate instruments	X	X
9)	Dissassemble	X	X

**Table 2:** Sensors and detectors on the calorimeter/combustor used to measure various physical parameters. The detector types are followed by their corresponding outputs

Sensor or detector	Output
Temperature:	
Thermocouples: Type T and Type K	microvolts
Quartz crystal thermometers [11]	frequency
Gas flow rate:	
Rotameters [9],[13]	volts
Differential temperature mass flow meters [8]	volts
Pressure:	
Absolute manometer [17]	volts
Differential manometers [18]	volts
Gas composition:	
Cooled-mirror humidity analyzer [14]	volts
Fixed wavelength infrared spectrometers [16]	millivolts
Scanning infrared spectrometer [15]	characters
Flame ionization detector hydrocarbon analyzer [12]	millivolts
Zirconium dioxide EMF cell oxygen analyzers [10],[19]	volts

**Table 3:** Tasks performed by our combustion calorimetry data acquisition system and their execution modes

Task	Foreground	Background
Instrument control		
Initialize devices	X	X
Select data input channels	X	
Trigger digitizers	X	
Data collection		
Voltage input	X	
Frequency input	X	
Serial input	X	
Data recording		
Store data on mass storage device	X	
Print data on hard copy device		X
Data calculations		
Convert data to physical quantities		X
Derive quantities from the data		X
Video display		
Show raw data voltage readings		X
Show calculated quantities		X
Plot data as a function of time		X
Show experiment control menus		X
Parameter input		
Reference temperature		X
Burn starting time		X
Scale values for plot routine		X
Software initialization		X
Error handling	X	

## Footnotes

1 Company products are identified to adequately specify the appartus. In no case does such identification imply recommendation or endorsement by the National Bureau of Standards, nor does it imply that the products are necessarily the best available for the purpose.

## Glossary

ASCII: Derived from the American Standard Code for Information Interchange. A seven- or eight-bit code used to represent alphanumeric characters.
BASIC: Derived from Beginners All-purpose Symbolic Instruction Code. A high-level programming language with a small repertoire of commands and a simple syntax. Fluke BASIC has an expanded instruction set for instrument control and other enhanced features.
Driver: a small program that controls external devices or interrupts.
Flag: any of various types of indications used for identification that signifies the occurrence of some condition.
Foreground: a program or process of high priority that utilizes machine facilities as needed with less critical, background, work performed in the otherwise unused time.
FORTRAN: Derived from formula translator. A high-level language used mostly for scientific or algebraic applications.
Interrupt: to stop a running program in such a way that it can be resumed at a later time, and in the meanwhile permit some other action to be performed.
Modular programming: the construction of a computer program from a collection of modules, each of workable size, whose interactions are rigidly restricted.
Module: a distinct and identifiable unit of a computer program dedicated to a particular process.
Multiprogramming: the interleaved execution of two or more programs by a computer, in which the central processing unit executes a few instructions from each program in succession.
Overlay: a technique for bringing routines into high-speed storage from some other means of storage during processing, so that several routines will occupy the same storage location at different times; an overlay is used when the total storage requirements for instructions exceed the available main storage.
Pascal: a high-level language which emphasizes structured programming.
Process: a set of instructions, data, and control information capable of being executed by the central processing unit of a computer in order to accomplish some purpose; in a multiprogramming environment, processes compete with one another for control of the central processing unit.
RAM: random-access memory.
Scheduling mechanism: a systematic method of determining the order in which processes will be performed by a computer system.
Structured programming: the use of program design and documentation techniques that impose a uniform structure on all computer programs. In another sense, it is an approach to programming in which only three constructs are employed for governing flow of control through the program. These three constructs allow for sequential, conditional, and iterative control flow.
True subroutine: a section of computer software which may be developed separately from the calling program, have parameters exchanged between it and the calling routine, and have local variables not accessible from the other program segments.
Virtual memory: a combination of primary and mass storage memories that can be treated as a single memory by programmers because the computer itself translates a program or virtual address to the actual hardware address.
